# 1 Million Segmented Red Blood Cells With 240 K Classified in 9 Shapes and 47 K Patches of 25 Manual Blood Smears

**DOI:** 10.1038/s41597-024-03570-z

**Published:** 2024-07-02

**Authors:** Ahmed Elsafty, Ahmed Soliman, Yomna Ahmed

**Affiliations:** 1 PathOlOgics, LLC, Cairo, Egypt; 2 Department of Computer Science and Artificial Intelligence, Faculty of Engineering and IT, British University in Dubai (BUiD), Dubai, United Arab Emirates

**Keywords:** Image processing, Machine learning, Anaemia, Laboratory techniques and procedures, Myeloproliferative disease

## Abstract

Around 20% of complete blood count samples necessitate visual review using light microscopes or digital pathology scanners. There is currently no technological alternative to the visual examination of red blood cells (RBCs) morphology/shapes. True/non-artifact teardrop-shaped RBCs and schistocytes/fragmented RBCs are commonly associated with serious medical conditions that could be fatal, increased ovalocytes are associated with almost all types of anemias. 25 distinct blood smears, each from a different patient, were manually prepared, stained, and then sorted into four groups. Each group underwent imaging using different cameras integrated into light microscopes with 40X microscopic lenses resulting in total 47 K + field images/patches. Two hematologists processed cell-by-cell to provide one million + segmented RBCs with their XYWH coordinates and classified 240 K + RBCs into nine shapes. This dataset (Elsafty_RBCs_for_AI) enables the development/testing of deep learning-based (DL) automation of RBCs morphology/shapes examination, including specific normalization of blood smear stains (different from histopathology stains), detection/counting, segmentation, and classification. Two codes are provided (Elsafty_Codes_for_AI), one for semi-automated image processing and another for training/testing of a DL-based image classifier.

## Background & Summary

The complete blood count (CBC) is a frequently used laboratory test that ranks among the top four tests in terms of both volume and revenue in various countries, such as the U.S., Malaysia, India, Kenya, and Nigeria^1^. The findings of a CBC test are useful in most medical and surgical specialties, including cardiology and psychiatry^2,3^. Furthermore, CBC test results need interpretation and correlation with other medical tests and clinical findings in up to 75% of cases. The hematologists or pathologists perform a manual/visual examination of blood smears for around 20% of the CBC tests. This process starts with spreading a thin layer of blood (10–50 µL) on a glass slide, staining it to highlight different intracellular structures, and then using light microscopes or digital pathology systems to review and examine red blood cells (RBCs), white blood cells (WBCs), and platelets.

In most labs, the commonly used manual preparation of smears can lead to unsuitable regions for examination on the smears. Choosing the appropriate areas relies on assessing the balance between individual and overlapping RBCs, preferring fields with fewer overlapping cells for precise examination and counting. Staining is a complicated process that is influenced by technical, sample-related, and medical factors, resulting in variations in the context of the image^4,5^. Whole slide images (WSIs) produced by digital pathology scanners are becoming increasingly popular among pathologists, pathology departments, and researchers. The variability in staining poses a challenge for both pathologists and deep learning-based (DL) automated systems, and optical scanning introduces its own set of variations and distortions^6–8^.

**Fig. 1:**
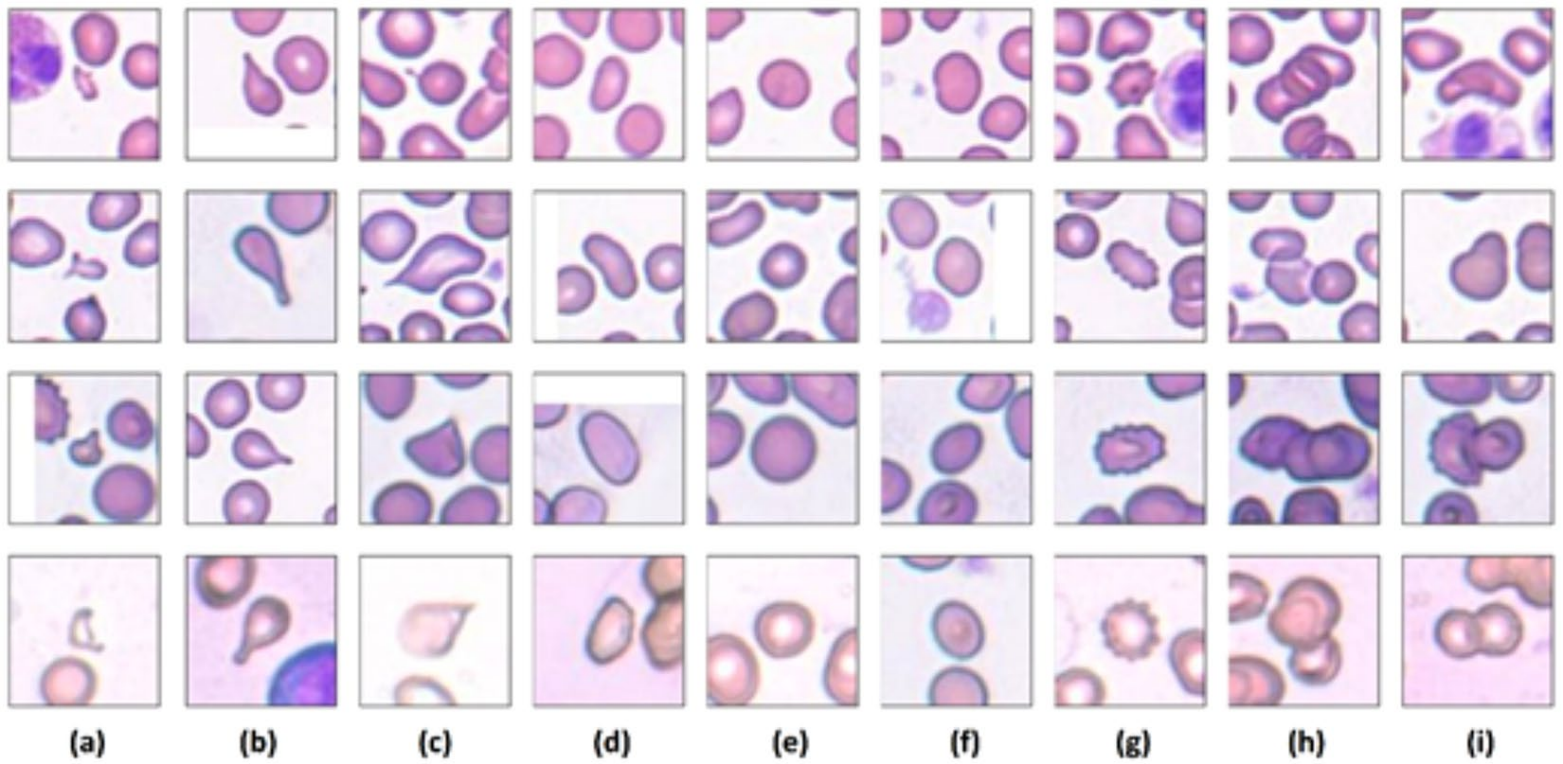
Examples of cropped RBCs images with perfect cellular centralization within the frame. (**a**) Fragmented RBCs, (**b**) Teardrop-shaped RBCs, (**c**) False Teardrop-shaped RBCs/Angled cells, (**d**) Ovalocytes, (**e**) Normal/rounded RBCs, (**f**) Borderline Ovalocytes, (**g**) Burr cells, (**h**) Three-overlapping RBCs, and (**i**) Two-overlapping RBCs. Samples in each row were obtained from each of the four provided imaging sources, with the top derived from source 1 and the bottom from source 4.

The aim of the provided dataset in this work (Elsafty_RBCs_for_AI)^9^ and the codes (Elsafty_Codes_for_AI)^10^, which are freely accessible at the Figshare data repository, is to facilitate the development and testing of a DL-based application for automated examination and reporting of RBCs morphology/shapes in percentages. Such an application is supposed to be capable of working with commonly used manually prepared and stained blood smears without necessitating prior standardization of the staining or smearing procedures. The provided 47 K + field images/patches from 25 different slides/patients are useful for developing and testing DL-based specific normalizers for blood smear stains, where there is a deficit and all prior arts/solutions in histopathology stains normalization are not applicable due to the different nature and results of the stains used. Furthermore, the provided one million + 80 × 80 pixels cropped images from the field images/patches containing segmented RBCs at their centers, along with the segmentation masks and the XYWH coordinates of the RBCs contours, enhance the development of DL-based segmenters and detectors. Moreover, the classified 240 K + images of RBCs enable the development of DL-based classifiers working on the real RBCs size, which is critical, without resizing. The provided RBCs classes are normal/rounded RBCs, ovalocytes (oval or egg-shaped), borderline ovalocytes (between rounded and frank oval), burr cells (crenated), schistocytes/fragmented RBCs, teardrop-shaped RBCs, two-overlapped RBCs, three-overlapped RBCs, and angled cells that contain false/artifact teardrops, schistocytes/fragmented RBCs and ovalocytes. Please note that RBCs shapes/classes which have alternative technological or laboratory tests for identification or confirmation, such as sickle cells and bite cells, were not included in this study. However, examples of cells with similar features to them were included in our class “angled cells.” Examples of cropped RBCs images are shown in Fig. 1.

The presence of schistocytes/fragmented RBCs or teardrop-shaped RBCs is medically significant as it is commonly associated with serious medical conditions. Schistocytes/fragmented RBCs are defined as RBCs that are smaller than half the average normal/rounded RBCs size and/or irregularly shaped fragments with sharp, angular, or jagged edges. Identifying these cells is the most reliable indicator to confirm the diagnosis of diseases such as hemolytic anemias, thrombotic thrombocytopenic purpura (TTP), and disseminated intravascular coagulation (DIC). However, reporting schistocytes/fragmented RBCs in TTP and DIC can be a challenge due to their infrequency in hematology labs; furthermore, the cutoff for significant presence in these two serious diseases is just above 1.0–1.5% of the total RBCs, increasing the risk of overlooking them^11,12^. Crucially, in cases of critical thrombocytopenia where the platelet count is less than 20 K/µL, platelet transfusion may be necessary, but this intervention can be life-threatening in TTP and DIC^13^. Therefore, identifying and counting schistocytes/fragmented RBCs is critical for the accurate diagnosis and management of patients with associated medical conditions.

Increased teardrop-shaped RBCs above 2.0–4.0% in adults can be indicative of bone marrow fibrosis caused by bone marrow cancers, and in non-cancerous conditions, rushed erythropoiesis/production of blood to compensate for severe anemia is the differential diagnosis. While in normal persons, the true teardrop-shaped RBCs are less than 0.5%. Currently, manual, or DL-based visual examination is the only way to identify teardrop-shaped RBCs^14^. It is essential to differentiate between true teardrop-shaped RBCs, which have a single blunt protrusion, and false ones that have sharp surface projections without necks or have more than one blunt protrusion. Mechanical stress during blood smear preparation often leads to the formation of false teardrop shapes, primarily at the outer edges of the blood film^15^.

Ovalocytes are a type of RBCs that have an abnormal oval shape. The presence of ovalocytes exceeding 5.0–10.0% of the total RBCs is associated with almost all types of anemia or erythrocytosis. They may display elongation and/or a pear shape, but without any blunt or sharp surface protrusions. Occasionally, they can also appear in normal blood smears due to mechanical deformation during preparation, though at a low frequency.

The burr cells have uneven surfaces with several small notches and protrusions. Likewise, no technological substitute currently exists for the visual recognition of burr cells, which tend to elevate under conditions of dehydration, such as in cases of renal failure or dehydrated neonates. Alternatively, in situations lacking medical justification, the presence of burr cells may arise due to the extended drying of smears during the manual staining procedure.

In comparison with prior works, including DL-based approaches and publicly available or locally-used RBCs datasets^16–22^, none has been created and reviewed directly by senior hematologists at the cell-by-cell level, nor provided comprehensive work to differentiate between true and false/artifact schistocytes/fragmented RBCs, ovalocytes, and teardrop-shaped RBCs, which are classes that lack technological assistance/confirmation alternative to visual examination. Additionally, none has utilized or created more than 24 K annotated RBCs, which is a fraction of the provided annotated/labelled cells in this work. Furthermore, no study has utilized four integrated cameras alongside microscopes to enrich diversity. Moreover, none has been designed to enable end-to-end automated examination of such clinically significant RBCs morphology/shape classes.

## Methods

### Sample preparation and imaging

**Fig. 2:**
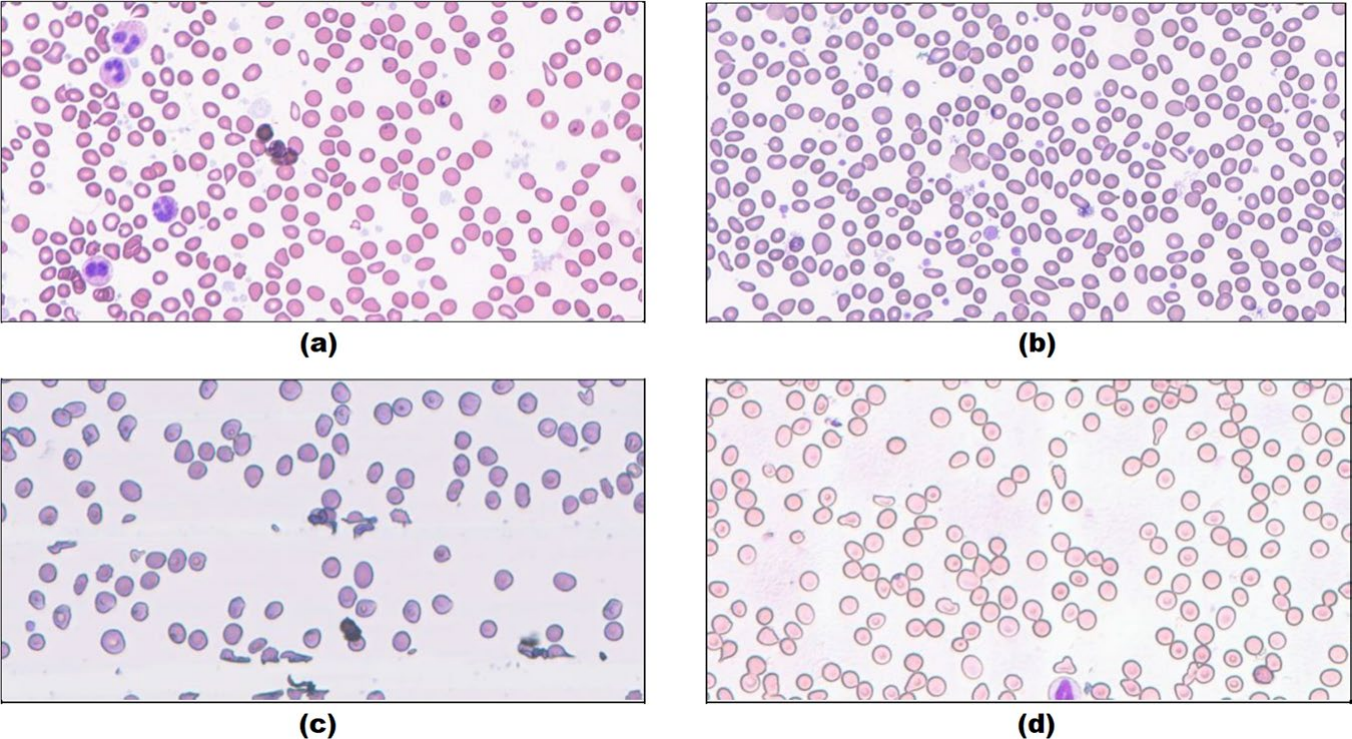
Examples of field images/patches from the four different imaging sources. (**a**) from source number one, (**b**) from source number two, (**c**) from source number three, and (**d**) from source number four. The patches obtained from the first and second sources were found to have the best imaging and staining quality, whereas those obtained from the third source exhibited relatively lower staining quality, and those from the fourth source showed relatively lower imaging or focus quality.

Blood smears were collected with written informed consents and the participants consented to the open publication of the data. This study was conducted with approval from the independent Research Ethics Committee of the Faculty of Medicine at Zagazig University, independent from the authors of this work, under ZU-IRB#:11225-24-10-2023. The samples were collected and smears were manually prepared and stained using Wright staining within the typical framework of clinical care. The inclusion criteria comprised patients suspected to have primary myelofibrosis (PMF) of the bone marrow, with confirmation based on a blood smear review revealing the presence of true teardrop-shaped RBCs. To ensure classification under the same conditions and collection of samples for every RBCs class from each patient, smears not containing all the nine predefined classes were excluded. Based on these inclusion and exclusion criteria, 25 blood smears, each obtained from a different patient, were found eligible for selection. The smears were categorized into four groups to enable the use of distinct digital cameras integrated with separate standard light microscopes for capturing field images/patches of the smears/slides. The type of the four cameras used was LCMOS02000KPB with a resolution of 1600 × 1200 pixels and a pixel size of 3.2 × 3.2 pixels/μm, manufactured by Nanjing Amada Instruments Co., Ltd in China. Utilizing the 40X microscopic objective lenses across all microscopes, in addition to the fixed 10X visual lenses, resulted in a total magnification power of 400X. Each microscopic field image was captured and used to crop a central rectangular image/patch with a consistent size of 1076 × 535 pixels. This specific size was chosen to align with the dimensions of the large touchscreen displays utilized for data processing. If a cropped image was incomplete due to any mistake, the remaining area was filled with a white background to ensure the completion of the image without overlapping with the adjacent fields. The dataset summary for each slide/patient, RBCs class, and camera-microscope source is presented in Table 1. The first camera-microscope was used on slides/patients numbered 1, 5, 6, 8 and 25. The second encompassed slides/patients numbered 2, 3, 4, 7, 9 and 11. The third comprised slides/patients numbered 14, 15, 19, 20, 22, 23 and 24. While the fourth contained slides/patients numbered 10, 12, 13, 16, 17, 18 and 21. A simple motorizing control unit was used for systematic smear navigation without any field repetition or overlap. The field images/patches obtained from the first and second cameras were found to have the best resolution and staining quality, whereas those obtained from the third exhibited relatively lower staining quality, and those from the fourth showed relatively lower resolution or focus quality. There was a total of 47 K + field images/patches from the 25 different slides/patients, comprising both suitable and non-suitable patches for RBCs examination. The determining factor for suitability was the presence of 100–300 individual RBCs among a few overlapping cells. Examples of field images/patches from different sources are shown in Fig. 2.

**Table 1:** The total segmented cells in each slide/patient and the tally of each RBCs class within every slide/patient across each camera-microscope source.

Camera-Microscope Source	Slide	Total Segmented Cells Per Slide	Rounded	Ovalocyte	Fragmented	Two Overlap	Three Overlap	Burr Cells	Teardrops	Angled Cells	Borderline Ovalocyte	Total Labelled Cells Per Slide
	Total	1,003,813	46,338	55,073	7,186	31,360	15,577	8,948	16,298	24,187	35,540	240,507
	Max per Slide	78,892	4,687	3,900	774	2,522	1,710	1,260	2,939	4,749	2,600	17,459
	Avg per Slide	40,153	1,854	2,203	287	1,254	623	358	652	967	1,422	9,620
	Min per Slide	15,336	797	981	30	421	178	16	26	69	689	5,395
1	1	54,507	4,687	3,156	486	1,698	586	750	2,579	1,704	1,813	17,459
2	2	71,068	940	2,593	432	1,690	1,176	216	551	1,875	1,641	11,114
2	3	35,557	2,098	1,664	760	1,183	703	391	1,368	405	1,349	9,921
2	4	51,982	919	2,371	730	590	376	316	756	359	1,415	7,832
1	5	39,127	1,575	1,618	317	1,465	1,003	302	479	1,080	1,313	9,152
1	6	59,345	1,654	1,555	359	1,954	814	338	444	960	1,700	9,778
2	7	52,243	797	3,820	602	2,401	1,710	504	1,349	4,749	1,152	17,084
1	8	78,892	972	3,514	463	2,280	1,318	379	2,939	3,501	1,140	16,506
2	9	53,279	1,314	3,900	548	1,617	527	331	986	1,490	1,817	12,530
4	10	44,730	1,277	3,417	526	1,163	566	371	1,175	1,223	1,337	11,055
2	11	60,139	1,193	3,852	774	2,522	1,018	183	1,427	892	2,175	14,036
4	12	31,917	2,918	1,037	50	810	444	42	153	81	854	6,389
4	13	20,845	2,623	981	54	864	452	35	141	69	1,092	6,311
3	14	33,331	1,967	2,444	30	1,402	445	639	243	236	968	8,374
3	15	28,539	1,340	1,668	45	421	465	830	187	193	910	6,059
4	16	42,635	2,646	2,125	138	829	398	25	176	292	1,086	7,715
4	17	33,375	1,983	1,189	38	890	352	16	53	185	689	5,395
4	18	34,466	2,494	1,760	127	1,104	493	47	144	2,096	1,083	9,348
3	19	39,453	1,818	1,320	30	860	436	1,260	130	227	886	6,967
3	20	15,648	2,223	2,017	36	1,138	427	662	59	187	2,054	8,803
4	21	23,055	1,441	985	39	887	535	492	79	163	1,269	5,890
3	22	31,973	2,755	1,007	47	603	241	39	26	94	2,600	7,412
3	23	15,482	1,444	2,176	88	1,001	516	251	72	209	1,896	7,653
3	24	15,336	1,526	2,061	126	796	178	350	49	471	1,681	7,238
1	25	36,889	1,734	2,843	341	1,192	398	179	733	1,446	1,620	10,486

Samples for every class were collected from each slide/patient.

### Images segmentation

**Fig. 3:**
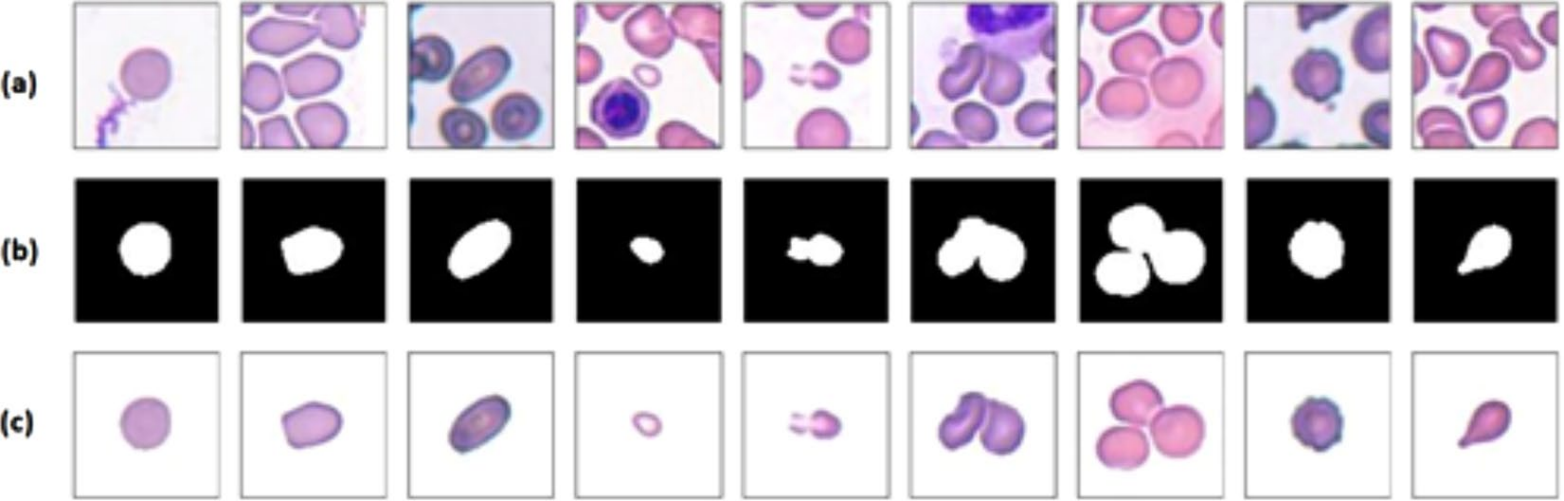
Examples of cells with their corresponding segmentation masks. (**a**) the cropped RBCs images, (**b**) the corresponding binary semantic segmentation ground-truth masks, and (**c**) the segmented RBCs images.

The hematologists developed their own semi-automated algorithms for image segmentation utilizing their concurrent Hematology and Software Engineering experience (please see Elsafty_Code_1; segment & localize using a pen^10^). This algorithm relied on manually tracing the borders of each cell using a digital pen tool on a big touchscreen display showing field images/patches. This process generated a ground-truth binary semantic segmentation mask and determined the bounding box coordinates (XYWH) for each cell. The cell contours were padded to ensure perfect centering within each image, maintaining a consistent size of 80 × 80 pixels for cropping. This fixed size is crucial to prevent the need for image resizing, as resizing could lead to misclassification of schistocytes/fragmented RBCs. If there was not enough space on the patch for an 80 × 80 pixels image due to the proximity of the cell to the borders, the remaining area was filled with a white background to complete the image. Cells situated along the borders that were truncated by the edge of the patch were excluded to prevent the risk of misclassification. This precaution was taken because the obscured section of the cell could impact the precise identification of the cell. The algorithm produced three 80 × 80 pixels images for each cell: the generated mask, the cropped image, and the segmented image. Each of these images adheres to a standardized naming convention, starting with the slide/patient number, followed by the patch number, and concluding with the (XYWH) coordinates. By utilizing this semi-automated approach, the hematologists were able to eliminate the attached background noise closely resembling the cellular colors caused by staining precipitates from the cells, as well as remove any attached WBCs or platelets. Moreover, it allowed for accurate segmentation of cells displaying empty areas due to mechanical stress during the spreading/smearing process or complications during imaging. This prevented the erroneous segmentation of a single cell into two separate schistocytes/fragmented RBCs. Examples of cells with their masks are shown in Fig. 3.

### Images review and labelling

Each cropped image along with its segmented image in the dataset for classification, underwent a comprehensive visual assessment by the two certified senior specialists in Hematology. Multiple rounds of comprehensive reviews and corrections of labels and segmentations were conducted until an expected level of high quality was attained. The labelling criteria were crafted to emphasize clinically significant RBCs classes, where visual examination is currently considered exclusive and unassisted by other technological solutions. Identifying ovalocytes through current manual/visual methods is subjective. This inherent subjectivity and absence of automated measures might account for the broad cutoff range (above 5–10%) observed in cases of anemia or erythrocytosis. To address this issue, aspect ratio calculations was utilized for preliminary classification. To calculate the long axes, three different methods were used, and the maximum result was considered. The first method involved rotating each cell mask image to an upright position by applying the rotation angle of the fitted ellipses in the opposite direction; then, the longer dimension of the corresponding upright bounding box was calculated. The second method involved applying a minimum enclosing rectangle to calculate its longer dimension, and the third method involved applying a minimum enclosing circle for the same purpose. For calculating the short axes, the shorter dimensions of the minimum enclosing rectangles were calculated and used. This combined approach was observed to yield more consistent performance when compared with individual methods. Measuring the distance between the two farthest points on the surface could result in an overestimation of the long axis, while relying solely on the minimum enclosing rectangle may lead to an underestimation of the long axis, especially in the case of rotated cells at 45 degrees. This measurement helped to preliminary distinguish between normal/rounded RBCs, borderline ovalocytes, and ovalocytes (1.0, 1.2, and 1.4 aspect ratios, respectively). The determination of these aspect ratios cutoffs was inspired from a blog discussing diamond measurements and shapes, with a focus on fine details and precision^23^. There is a separate class named “angled cells.” This class contained numerous RBCs that exhibited similarities to schistocytes/fragmented RBCs, ovalocytes, and teardrop-shaped RBCs but were in fact false representations of these classes. Identification of overlapping RBCs can be challenging given that upper cells might mask crucial parts of the overlapped cells, leading to potential misclassification of the overlapped. Therefore, there is no need to assume or predict the actual types of the overlapping RBCs. They were included in separate classes just to enable the classifiers to differentiate individual cells from them (junk classes).

## Data Records

(Elsafty_RBCs_for_AI) dataset^9^ is freely accessible at the Figshare data repository and is systematically structured into 51 root directories. The first root directory (Elsafty_RBCs_for_Classification) consists of three primary folders: “Cropped images,” “Masks,” and “Segmented images.” Within each of these primary folders, there are nine subfolders, meticulously dedicated to each RBCs class, encompassing the following counts of cells: “Angled cells: 24,187”, “Borderline ovalocytes: 35,540”, “Burr cells: 8,948”, “Fragmented RBCs: 7,186”, “Ovalocytes: 55,073”, “Rounded RBCs: 46,338”, “Teardrops: 16,298”, “Three-overlapping RBCs: 15,577”, and “Two-overlapping RBCs: 31,360”. Each of the total 240,507 cells is represented by its own cropped image, mask, and segmented image. Samples for every class were collected from each slide/patient. Each one of the next 25 root directories (Elsafty_RBCs_for_Segmentation_and_Detection_Slide_1–25) consists also of three primary folders: “Cropped images,” “Masks,” and “Segmented images” corresponding to each slide/patient. There is a total of 1,003,813 segmented cells along with their masks and cropped images, the counts of segmented cells per slide/patient, sorted in ascending order, are as follows: 54507, 71068, 35557, 51982, 39127, 59345, 52243, 78892, 53279, 44730, 60139, 31917, 20845, 33331, 28539, 42635, 33375, 34466, 39453, 15648, 23055, 31973, 15482, 15336, and 36889. The total segmented cells in each slide/patient and the tally of each RBCs class within every slide/patient are presented in Table 1. The naming scheme for the cropped image, mask, and segmented image of every cell adheres to a consistent format, starting with the slide/patient number, followed by the unique patch/field number, and concluding with the (XYWH) coordination on the patch. All these images are conveniently stored in the lossless “.PNG” format. Each one of the remaining 25 root directories (Elsafty_RBCs_Slide_1–25) contains the field images/patches of a specific slide/patient. The names of the patches start with the respective slide/patient number, followed by the unique patch/field number. There is a total of 47,363 patches with 1076 × 535 pixels size from the 25 slides/patients, the counts of patches per slide/patient, sorted in ascending order, are as follows: 1690, 1709, 2697, 2400, 1905, 1803, 2964, 3049, 2464, 2080, 1894, 1852, 2590, 2263, 3328, 983, 1066, 935, 1131, 1237, 1874, 1393, 1277, 1199, and 1580.

The “Elsafty_Codes_for_AI,”^10^ contains two files:

1. Elsafty_Code_1; segment & localize using a pen.
2. Elsafty_Code_2; train & test a DL-based image classifier, using Google Colab. Please note that this code is specifically designed to be executed within the Google Colab environment.

## Technical Validation

The hematologists have developed and used their code to train DL-based image classification models using TensorFlow/Keras, (please see Elsafty_Code_2; train & test a DL-based image classifier using Google Colab^10^). During the training process utilizing a trainable EfficientNetB0 for transfer learning, all the segmented images for each class in their respective folders, sourced from the 25 slides/patients, were divided into six separate parts. One part was allocated for testing, the second for validation, and the remaining four parts for training. There were two options in the code: whether to shuffle the images randomly with a fixed seed before splitting or not. Shuffling ensured that the code split the dataset without allocating images from certain slides/patients to specific subsets. While useful for exploring data consistency, this approach was not reliable for generalizing performance. Conversely, without shuffling, the splitting resulted in better performance generalization because validation and testing were conducted on different cases. The initial learning rate and batch size used during training were 4e-6 and 32, respectively. Adam optimizer, SparseCategoricalCrossentropy loss and SparseCategoricalAccuracy metric were implemented. To prevent overfitting, common augmentation techniques including full rotation range (up to 360 degrees), vertical flipping and horizontal flipping were employed. While color manipulation, rescaling, shearing, shifting, zooming, and resizing were avoided. After developing a model with no shuffling of the dataset before splitting, the evaluation revealed the following results for overall specificity, F1 score, and accuracy: 0.9986, 0.9884, and 0.9974, respectively, indicating data consistency and quality. Subsequently, new synthetic images were generated from the real-world images using extensive color manipulation by randomly overlaying six main colors ((255,0,0), (0,255,0), (0,0,255), (0,255,255), (255,0,255), (255,255,0)) with varying degrees of transparency (alpha) and intensity (beta), ranging from 0.5 to 1.1, before utilizing the masks again to restore the white background. The same model was then evaluated on the new synthetic images. This revealed the following results for overall specificity, F1 score, and accuracy: 0.9833, 0.8667, and 0.9704, respectively. These results indicated the potential usefulness of stain normalizers to reduce performance fluctuations and induce generalizability. Please see Tables 2, 3 for evaluation details, including the confusion matrix, individual class metrics, and overall performance metrics, where the top portions of these tables correspond to evaluation on real-world images and the bottom portions correspond to evaluation on synthetic color-manipulated images. To further investigate the effect of normalization lack, the entire dataset for segmentation was classified using the same classifier on both the original and its synthetic color-manipulated versions. Without normalization, there is a potential risk of false increase of schistocytes/fragmented RBCs and angled cells with false decrease of teardrop-shaped RBCs, especially when the staining is weak and faint. Please refer to Table 4, where within each result box, the left side corresponds to the evaluation on the real-world images, while the right side corresponds to the evaluation on the synthetic color-manipulated images.

**Table 2:** The confusion matrix of the model developed using the full dataset for classification.

	Rounded	Ovalocytes	Fragmented	Two Overlap	Three Overlap	Burr Cells	Teardrops	Angled Cells	Borderline Oval.
Rounded	46,296	—	—	1	—	—	—	6	35
Ovalocytes	1	54,779	4	41	—	3	18	64	163
Fragmented	3	23	7,076	—	—	5	2	72	5
Two Overlap	1	45	—	30,620	553	18	20	78	25
Three Overlap	—	2	—	305	15,270	—	—	—	—
Burr Cells	6	43	1	42	—	8,765	—	73	18
Teardrops	—	60	2	21	—	2	16,088	125	—
Angled Cells	20	142	29	150	—	22	125	23,634	65
Borderline Oval.	246	56	1	2	—	—	—	41	35,194
	**Rounded**	**Ovalocytes**	**Fragmented**	**Two Overlap**	**Three Overlap**	**Burr Cells**	**Teardrops**	**Angled Cells**	**Borderline Oval**.
Rounded	42,293	—	823	20	—	1	—	61	3,135
Ovalocytes	—	51,763	1,542	576	—	8	11	495	650
Fragmented	15	44	6,962	—	—	17	2	123	12
Two Overlap	7	1,589	187	25,091	2,464	97	60	1,632	217
Three Overlap	1	47	43	4,152	11,307	3	—	8	—
Burr Cells	75	352	191	506	12	6,670	—	864	273
Teardrops	—	464	526	410	—	1	12,218	2,665	—
Angled Cells	59	938	1,711	1,231	4	25	82	19,299	814
Borderline Oval.	817	698	803	256	—	2	—	226	32,721

The top portion corresponds to evaluation on real-world images and the bottom portion corresponds to evaluation on synthetic color-manipulated images.

**Table 3:** The evaluation details include the individual class metrics and overall performance metrics.

	True Positive	True Negative	False Positive	False Negative	Sensitivity	Specificity	Precision	F1 Score	Accuracy
Rounded	46,296	193,892	277	42	0.9991	0.9986	0.9941	0.9966	0.9987
Ovalocytes	54,779	185,063	371	294	0.9947	0.9980	0.9933	0.9940	0.9972
Fragmented	7,076	233,284	37	110	0.9847	0.9998	0.9948	0.9897	0.9994
Two Overlap	30,620	208,585	562	740	0.9764	0.9973	0.9820	0.9792	0.9946
Three Overlap	15,270	224,377	553	307	0.9803	0.9975	0.9651	0.9726	0.9964
Burr Cells	8,765	231,509	50	183	0.9795	0.9998	0.9943	0.9869	0.9990
Teardrops	16,088	224,044	165	210	0.9871	0.9993	0.9898	0.9885	0.9984
Angled Cells	23,634	215,861	459	553	0.9771	0.9979	0.9809	0.9790	0.9958
Borderline Oval.	35,194	204,656	311	346	0.9903	0.9985	0.9912	0.9908	0.9973
Total	237,722	1,921,271	2,785	2,785	0.9884	0.9986	0.9884	0.9884	0.9974
	**True Positive**	**True Negative**	**False Positive**	**False Negative**	**Sensitivity**	**Specificity**	**Precision**	**F1 Score**	**Accuracy**
Rounded	42,293	193,064	974	4,040	0.9128	0.9950	0.9775	0.9440	0.9791
Ovalocytes	51,763	181,194	4,132	3,282	0.9404	0.9777	0.9261	0.9332	0.9692
Fragmented	6,962	227,370	5,826	213	0.9703	0.9750	0.5444	0.6975	0.9749
Two Overlap	25,091	201,876	7,151	6,253	0.8005	0.9658	0.7782	0.7892	0.9442
Three Overlap	11,307	222,330	2,480	4,254	0.7266	0.9890	0.8201	0.7705	0.9720
Burr Cells	6,670	231,274	154	2,273	0.7458	0.9993	0.9774	0.8461	0.9899
Teardrops	12,218	223,932	155	4,066	0.7503	0.9993	0.9875	0.8527	0.9824
Angled Cells	19,299	210,134	6,074	4,864	0.7987	0.9719	0.7606	0.7792	0.9545
Borderline Oval.	32,721	199,747	5,101	2,802	0.9211	0.9751	0.8651	0.8922	0.9671
Total	208,324	1,890,921	32,047	32,047	0.8667	0.9833	0.8667	0.8667	0.9704

The top portion corresponds to evaluation on real-world images and the bottom portion corresponds to evaluation on synthetic color-manipulated images.

**Table 4:** The results of classifying the entire dataset for segmentation.

Total Individual Cells	Total Individual Cells	Rounded		Ovalocytes		Fragmented		Burr Cells		Teardrops		Angled Cells		Borderline Oval.	
	830,046	276,957		126,095		5,180		12,630		11,930		38,063		359,271	
Slide 1	47,733	28.4%	26.4%	16.1%	16.7%	0.8%	2.2%	2.4%	1.8%	4.5%	3.1%	4.6%	7.6%	43.3%	42.2%
Slide 2	54,351	24.7%	22.0%	20.1%	20.3%	0.9%	3.8%	1.3%	0.8%	1.2%	0.6%	7.8%	10.8%	44.0%	41.7%
Slide 3	28,429	27.2%	25.1%	20.1%	18.5%	1.8%	6.2%	2.5%	2.1%	3.1%	2.0%	4.2%	9.3%	41.2%	36.7%
Slide 4	48,343	29.1%	28.6%	20.2%	19.3%	1.1%	3.2%	2.9%	2.3%	2.3%	1.5%	3.2%	5.6%	41.2%	39.5%
Slide 5	30,927	47.1%	44.3%	7.9%	8.9%	0.3%	1.0%	1.1%	0.9%	0.9%	0.5%	2.6%	4.1%	40.0%	40.3%
Slide 6	47,766	41.7%	38.5%	9.0%	9.7%	0.6%	2.5%	0.8%	0.6%	0.7%	0.4%	4.6%	6.5%	42.6%	41.8%
Slide 7	45,981	25.6%	24.4%	16.2%	15.1%	0.3%	2.0%	0.5%	0.4%	2.1%	1.4%	8.5%	11.2%	46.7%	45.5%
Slide 8	58,694	25.5%	22.9%	16.8%	17.0%	0.4%	2.3%	0.5%	0.5%	2.2%	1.4%	7.7%	11.0%	46.9%	44.8%
Slide 9	48,097	31.8%	29.7%	17.4%	16.9%	0.5%	3.4%	0.8%	0.6%	1.0%	0.6%	3.9%	5.8%	44.7%	43.0%
Slide 10	28,970	28.1%	23.9%	18.3%	19.1%	0.7%	2.9%	2.3%	1.7%	1.6%	0.9%	5.3%	9.9%	43.8%	41.7%
Slide 11	49,899	29.6%	28.7%	17.9%	19.3%	0.4%	1.6%	0.5%	0.3%	1.7%	1.2%	2.2%	4.0%	47.8%	44.9%
Slide 12	26,348	52.8%	51.6%	6.0%	5.9%	0.3%	2.7%	0.1%	0.1%	0.5%	0.4%	1.0%	2.2%	39.3%	37.1%
Slide 13	15,482	42.8%	43.7%	9.6%	9.4%	0.5%	0.8%	0.4%	0.2%	0.6%	0.6%	2.6%	3.1%	43.5%	42.3%
Slide 14	25,908	21.6%	21.5%	23.4%	22.5%	0.2%	0.7%	4.7%	3.0%	1.3%	0.9%	4.8%	8.4%	43.9%	42.9%
Slide 15	23,955	24.5%	26.3%	21.2%	19.5%	0.3%	0.9%	4.8%	2.2%	1.0%	0.3%	3.8%	5.6%	44.4%	45.1%
Slide 16	39,098	35.1%	32.7%	14.1%	12.9%	0.7%	6.4%	0.1%	0.1%	0.6%	0.3%	2.4%	4.9%	47.0%	42.7%
Slide 17	28,547	48.7%	45.3%	8.8%	7.9%	1.1%	7.9%	0.1%	0.1%	0.3%	0.2%	2.5%	5.7%	38.4%	33.0%
Slide 18	32,977	44.2%	39.3%	10.3%	9.0%	0.7%	6.5%	0.1%	0.1%	0.2%	0.1%	6.1%	7.5%	38.3%	37.6%
Slide 19	32,841	32.4%	33.5%	11.6%	11.0%	0.2%	0.7%	4.8%	2.3%	0.7%	0.3%	5.2%	6.8%	45.1%	45.4%
Slide 20	12,587	27.2%	27.6%	18.9%	18.7%	0.3%	0.3%	4.1%	3.8%	0.5%	0.5%	3.7%	4.2%	45.3%	44.9%
Slide 21	15,077	36.2%	39.3%	12.6%	11.7%	0.7%	2.0%	2.9%	2.5%	0.6%	0.5%	4.1%	4.6%	42.8%	39.5%
Slide 22	27,783	64.5%	62.8%	4.4%	4.1%	0.3%	1.5%	0.1%	0.1%	0.1%	0.1%	0.7%	1.1%	30.0%	30.3%
Slide 23	11,568	15.8%	16.7%	26.3%	26.1%	1.1%	1.2%	2.6%	2.1%	0.6%	0.6%	5.4%	6.7%	48.3%	46.6%
Slide 24	14,382	23.9%	25.0%	18.8%	18.3%	1.0%	1.2%	3.7%	2.8%	0.6%	0.5%	8.4%	9.9%	43.7%	42.3%
Slide 25	34,303	34.5%	32.3%	13.6%	13.2%	0.6%	2.0%	0.5%	0.3%	2.1%	1.5%	4.8%	6.3%	43.9%	44.3%

Within each result box, the left side corresponds to the evaluation on the real images, while the right side corresponds to the evaluation on the synthetic color-manipulated images.

To compare the quality of the segmented images and labelling from each of the four camera-microscope sources, four rotating leave-one-out classification experiments were conducted. In these experiments, all images from slides of a rotating source were excluded during training, and the trained model was then tested on these excluded images. For details and results, please see Table 5. Additionally, another 12 classification experiments were conducted using one rotating source for training and one of the remaining sources for testing. The details and results are displayed in Table 6. The findings of these experiments indicated inter-source variations with overall high labelling and segmentation quality.

**Table 5:** The details and results of the four leave-one-out classification experiments.

	Classification Model 1	Classification Model 2	Classification Model 3	Classification Model 4
Training Set Imaging Sources	2, 3, 4	1, 3, 4	1, 2, 4	1, 2, 3
Training Subset Images	118,084	111,993	125,334	125,602
Validation Subset Images	29,521	27,998	31,333	31,400
Training Accuracy	0.9799	0.9831	0.9857	0.9822
Validation Accuracy	0.9833	0.9874	0.9895	0.9870
Test Set Imaging Source	1	2	3	4
Test Set Images	63,381	72,517	52,506	52,103
Test Overall F1 Score	0.9814	0.9731	0.9154	0.9601

**Table 6:** The details and results of the 12 one-source-only classification experiments.

	Classification Model 5	Classification Model 6	Classification Model 7	Classification Model 8	Classification Model 9	Classification Model 10
Training Set Imaging Source	1	1	1	2	2	2
Training Subset Images	42,254	42,254	42,254	48,344	48,344	48,344
Validation Subset Images	10,563	10,563	10,563	12,086	12,086	12,086
Training Accuracy	0.9807	0.9807	0.9807	0.9754	0.9754	0.9754
Validation Accuracy	0.9841	0.9841	0.9841	0.9818	0.9818	0.9818
Test Set Imaging Source	2	3	4	1	3	4
Test Set Images	72,517	52,506	52,103	63,381	52,506	52,103
Test Overall F1 Score	0.9856	0.9876	0.9869	0.9826	0.9727	0.9808
	**Classification Model 11**	**Classification Model 12**	**Classification Model 13**	**Classification Model 14**	**Classification Model 15**	**Classification Model 16**
Training Set Imaging Source	3	3	3	4	4	4
Training Subset Images	35,004	35,004	35,004	34,735	34,735	34,735
Validation Subset Images	8,751	8,751	8,751	8,683	8,683	8,683
Training Accuracy	0.9766	0.9766	0.9766	0.9806	0.9806	0.9806
Validation Accuracy	0.9815	0.9815	0.9815	0.9847	0.9847	0.9847
Test Set Imaging Source	1	2	4	1	2	3
Test Set Images	63,381	72,517	52,103	63,381	72,517	52,506
Test Overall F1 Score	0.9641	0.9499	0.9618	0.9856	0.9841	0.9815

## Usage Notes

Regarding stain normalization, please note that in contrast to histopathology stains, which typically distinguish structures into two colors (red and blue) or three (red, black, and blue), stained blood smears exhibit at least seven significant colors for RBCs, WBCs, and platelets (red, orange, grey, deep purple, violet, light blue, and blue). Additionally, unlike the distinct shapes and sizes of RBCs, the color of RBCs is influenced by the context of the field image/patch and encompasses a range from red, pink, brown, yellow, orange, light violet, to even near bluish hues depending on the staining and imaging quality. However, the RBCs color is the closest to red and furthest from blue among the overall staining colors, while WBCs nuclei tend to exhibit the opposite pattern. While specific normalizers for blood smear stains could be useful, manipulation of RBCs shape and size should be avoided. Normalizers for blood smear stains should be comprehensively assessed in two or more ways. Firstly, through quantitative evaluation of their contribution to the classification and detection performances. Secondly, through qualitative visual inspection for potential artificial errors, such as the discoloration of small blue platelets into red, resulting in misclassification as schistocytes/fragmented RBCs. Conversely, discoloring red schistocytes/fragmented RBCs into blue could also lead to their misclassification as platelets.

In terms of RBCs detection, please note that for all field images/patches selected to generate dataset for segmentation, the overlapping RBCs occupying bounding boxes larger than 80 × 80 pixels and the RBCs touching the borders of the field images/patches were excluded, because truncated cells by the borders could be misclassified as schistocytes/fragmented RBCs. Please also note that the identification/classification of overlapped cells may not be accurate, as assuming the covered parts is not appropriate. Therefore, please count the individual cells and exclude the overlapping ones. The overlapping cells could be utilized to identify the appropriate field image/patch for examination. The required one thousand individual cells to calculate the percentage of each RBCs class could be collected from three to ten appropriate/suitable field images/patches. Additionally, the maximum percentages of schistocytes/fragmented RBCs and teardrop-shaped RBCs present in each of the used appropriate/suitable field images/patches should be highlighted. Furthermore, applying techniques such as non-maximum size suppression could be essential to avoid misclassification of cellular parts as schistocytes/fragmented RBCs. Moreover, please collect false positive images from the detector to create an “excluded junk class” to be used in classification training. Otherwise, falsely cropped non-RBCs images may be misclassified as RBCs.

## Data Availability

Please find the following codes under the root directory “Elsafty_Codes_for_AI,”^10^: 1. Elsafty_Code_1; segment & localize using a pen. 2. Elsafty_Code_2; train & test a DL-based image classifier using Google Colab.
